# Cortisol inhibits mTOR signaling in avascular necrosis of the femoral head

**DOI:** 10.1186/s13018-017-0656-2

**Published:** 2017-10-18

**Authors:** Yun Liao, Rui Su, Ping Zhang, Bo Yuan, Ling Li

**Affiliations:** 1 Department of Pharmacy, Tongren Hospital, Shanghai Jiao Tong University School of Medicine, Shanghai, 200336 China; 2 Department of Pharmacy, Shanghai Tenth People’s Hospital, Tongji University, Shanghai, 200072 China

**Keywords:** Avascular necrosis of the femoral head, Cortisol, mTOR, HIF

## Abstract

**Background:**

ANFH is a major health problem, to which long lasting and definitive treatments are lacking. The aim of this study is to study RNA alterations attributed to cortisol-induced ANFH.

**Methods:**

Rat models were stratified into three groups: in vitro group (*n* = 20) for molecular biological assays, control group (*n* = 3), and ANFH group induced using lipopolysaccharide and dexamethasone (*n* = 3). Bone marrow-derived endothelial progenitor cells (BM-EPCs) were extracted from the rats. An RNA expression array was performed on BM-EPCs, and enriched genes were subject to pathway analysis. In vitro studies following findings of array results were also performed using the isolated BM-EPCs.

**Results:**

Significant alterations in mammalian target of rapamycin (mTOR) and HIF signaling pathways were identified in BM-EPCs of ANFH. By applying cortisol and dexamethasone to BM-EPCs, significant changes in mTOR and HIF elements were identified. The alteration of HIF pathways appeared to be downstream of mTOR signaling. Glucocorticoid receptor (GR) expression was related to glucocorticoid-dependent mRNA expression of mTOR/HIF genes. mTOR-dependent angiogenesis but not anabolism was the target of GR in ANFH. Inhibition of mTOR signaling also induced apoptosis of BM-EPCs via CHOP-dependent DR5 induction in response to GR stimulation.

**Conclusion:**

Decreased mTOR signaling in response to GR stimulation leading to downregulated HIF pathway as well as increased apoptosis could be the pathophysiology.

**Electronic supplementary material:**

The online version of this article (10.1186/s13018-017-0656-2) contains supplementary material, which is available to authorized users.

## Background

Avascular necrosis of the femoral head (ANFH) is a major health problem [1]. The pathological process initiates at the loading region of the femoral head. Following microfracture of the necrotic trabeculae by stress, immediate restoration commences, which is however in most cases inadequate and the equilibrium often tilts towards symptomatic ANFH [2]. Though hip replacement stands an ultimate option, the metal articulation has many limitations [3, 4]. Treatment other than surgery for ANFH is inconclusive, mainly due to uncertainty of its etiology aside from trauma, the one associated with inherent alteration of bone tissue, most commonly caused by chronic alcohol intoxication or excessive steroid use [5].

ANFH caused by steroid is even more detrimental as the population is often diagnosed at a younger age. This is often a result of pharmacologic doses of steroids used to treat inflammatory or autoimmune disorders. Generally, it is considered that glucocorticoids have a dose-dependent effect on the skeleton, such that longer duration and higher doses of steroids are most likely to cause bone loss and fractures [6]. There are a variety of mechanisms proposed from fat embolism to the uncoupling remodeling unit. Ischemia to the femoral head is the consequent pathologic change as the femoral head itself is anatomically poorly vasculated. Despite those, there has been a dearth of evidence that steroid could directly inhibit angiogenic potential of the femoral head.

The aim of the current study is to systematically analyze the genetic alteration of the progenitor cells of the femoral head in model animals for steroid-induced ANFH. By focusing on pathway alterations that are less popularly studied, the results may shed light on novel insights on the pathophysiology of ANFH. The findings may further complete the comprehensive network of ANFH and provide targetable or drugable nodes, additive to the current treatment modalities.

## Methods

### Establishment of bone marrow-derived endothelial progenitor cells (BM-EPC)

The BM-EPCs were explanted from adult Wistar rats (8–10 w, 250–300 g), purchased from Shanghai Experimental Animal Center, Chinese Academy of Sciences. Animals were fed processed per corresponding regulations of Shanghai Tenth People’s Hospital (Document No. SYXK 2014-0026). The animal ethics were reviewed by the local institutional review board referencing the Guide for Care and Use of Laboratory Animals published by the National Institutes of Health (Document No. SCXK 2013-0016). Rats were stratified into three groups after 1 week of feeding: in vitro group (*n* = 20) for subsequent molecular biological assays including western blotting and flow cytometry, control group (*n* = 3), and ANFH group (*n* = 3); the latter of which was induced using a combination of lipopolysaccharide (LPS, *Escherichia coli* serotype 055: B5; Sigma-Aldrich, St Louis, MO, USA) and dexamethasone (DEX, D1756-1G, Sigma-Aldrich, St Louis, MO, USA). Explantation of BM-EPCs was per previous report [7]. Cells for microarray were processed per protocol below. Cells for in vitro study were treated with reagents or interfered with shRNA after regular culture for 7 days. Reagents, doses, and time points used in the in vitro studies were cortisol (COR, 1.0 μM at 48 h), dexamethasone (DEX, 1.0 μM at 48 h), and rapamycin (RAPA, 10 μM at 48 h).

### LncRNA microarray

The Agilent murine lncRNA microarray (4×180K, Design ID: 049801) which also captured mRNA expression was used in this experiment. Total RNA was quantified by the NanoDrop ND-2000 (Thermo Scientific), and the RNA integrity was assessed using Agilent Bioanalyzer 2100 (Agilent Technologies). The sample labeling, microarray hybridization, and washing were performed based on the manufacturer’s standard protocols. Briefly, total RNA were transcribed to double strand cDNA, then synthesized into cRNA, and labeled with cyanine-3-CTP. The labeled cRNAs were hybridized onto the microarray. After washing, the arrays were scanned by the Agilent Scanner G2505C (Agilent Technologies). Feature Extraction software (version10.7.1.1, Agilent Technologies) was used to analyze array images to get raw data. Genespring were employed to finish the basic analysis with the raw data. To begin with, the raw data was normalized with the quantile algorithm, which computed the specified quantile of a sorted array of the exact reads. The probes that at least one condition out of two conditions have flags in “P” were chosen for further data analysis. Differentially expressed genes or lncRNAs were then identified through fold change as well as *P* value calculated with *t* test. The threshold set for up- and downregulated genes was a fold change ≥ 2.0 and a *P* value of ≤ 0.05. Afterwards, GO analysis (Gene Ontology, the framework for the model of biology, which defines concepts/classes used to describe gene function and relationships between these concepts) and KEGG analysis (Kyoto Encyclopedia of Genes and Genomes, which is a database resource for understanding high-level functions and utilities of the biological system) were applied to determine the roles of these differentially expressed mRNAs. Finally, hierarchical clustering was performed to display the distinguishable genes’ expression pattern among samples. Raw normalized data were presented in Additional file 1.

### Western blotting

Established protocol was reference in the current study [8]. Total protein of cell lysates was extracted and equal protein amount of 25 μg was loaded onto 10% sodium dodecyl sulfate polyacrylamide gel. Gels were subsequently transferred to nitrocellulose membrane. The membranes were blocked with 5% non-fat milk. Primary anti-mouse antibodies used were listed in Additional file 2: Table S1. Detection was performed using the ECL kit (electrochemiluminescence) from Thermo Scientific (Middletown, VA, USA). GAPDH (glyceraldehyde 3-phosphate dehydrogenase) was used as an internal standard.

### RNA interference

A standard protocol of the Fugene method was followed. Briefly, transfection was carried out on day 2 using 2 μg plasmid diluted in 200 μL of OptiMem (Gibco) and 6 μL of Fugene HD (Promega) per well. For shRNA construction, the target sequence was referenced from TRC (http://portals.broadinstitute.org/gpp/public/gene/details?geneId=2908), as follows: TRCN0000222129 as shRNA#1, and TRCN0000245003 as shRNA#2. Vectors with puromycin resistance were used and were transected in to cells using the Fugene system.

### Real-time RT-PCR

RNA was first converted to cDNA, which was then loaded to the ABI7500 apparatus using the SYBR Green system. Primers used were referenced from https://pga.mgh.harvard.edu/primerbank/ and were listed in Additional file 2: Table S2. GAPDH was chosen as internal reference. The expression of query genes was calculated according to internal references and was expressed as folds over the control group.

### Flow cytometry

The flow cytometry was used to study cell apoptosis. Briefly, cells treated or untreated were dyed using annexin V-fluorescein (BD Pharmingen, Pasig City, Philippines) and propidium iodide (PI) (BD). Samples were then analyzed with BD FACSCanto flow cytometer to determine percentages of apoptotic cells (including early apoptotic cells).

### Statistical analysis

The Prism Graphpad version 7 was used for statistical analyses. The two-tailed *t* test was used for comparison between results of distinctive assays. The *P* value of < 0.05 was accepted as statistically significant.

## Results

**Fig. 1:**
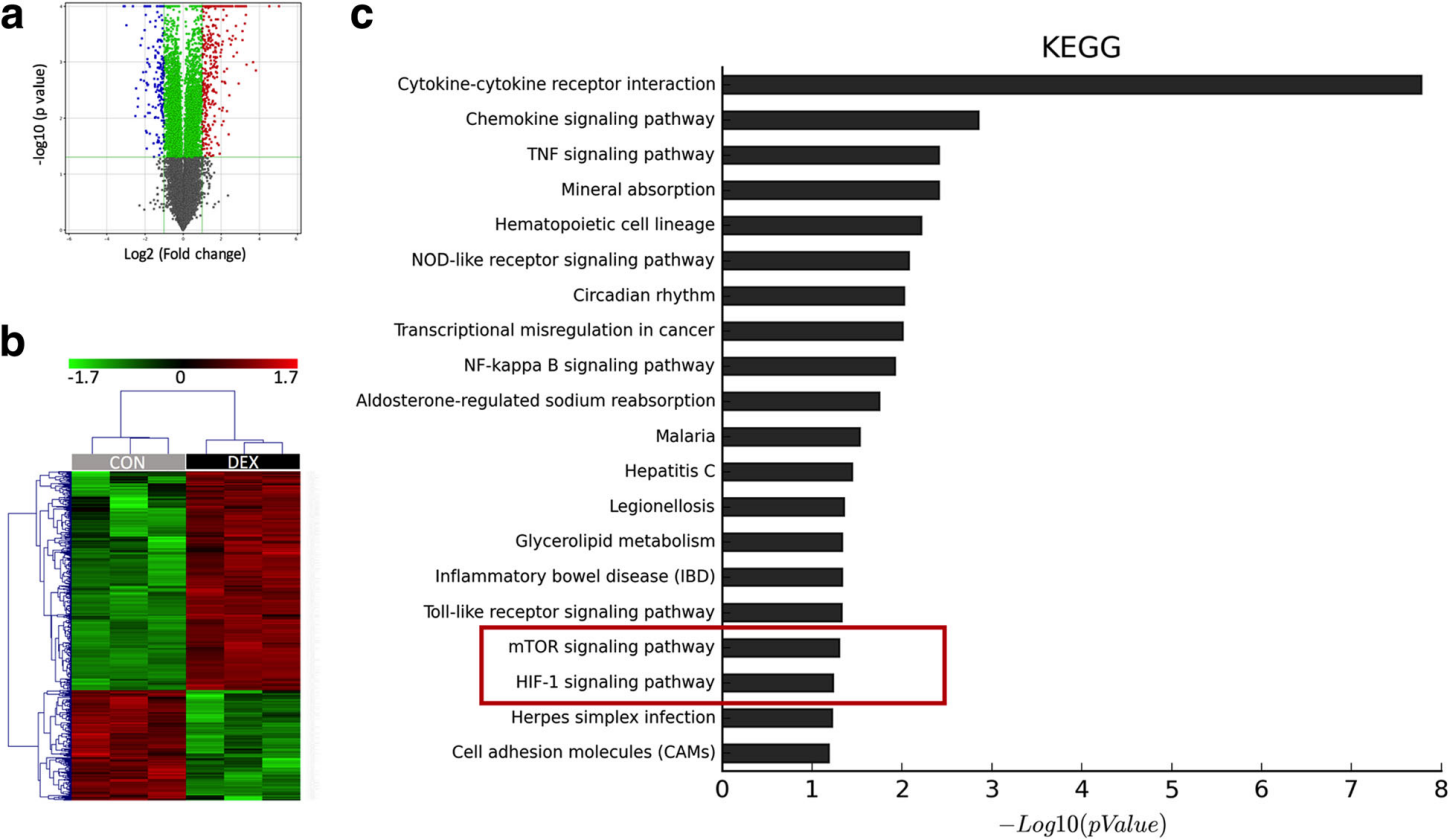
RNA microarray of BM-EPCs from ANFH and control mice revealing differentially expressed genes and pathway annotations. **a** Volcano plots showing significantly changed genes both upregulated and downregulated. **b** Heatmap showing clustered differentially expressed genes. **c** Pathway analysis showing pathway changes ranked by significant power

**Fig. 2:**
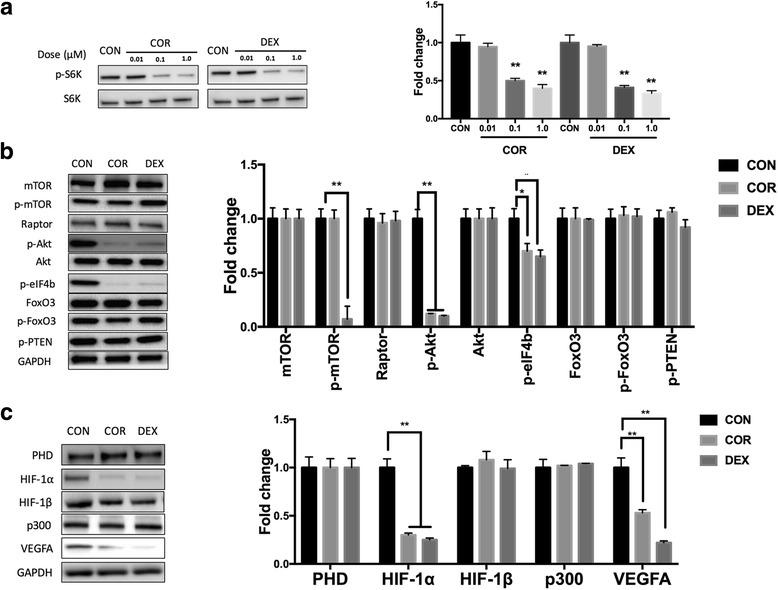
Glucocorticoids inhibited mTOR and HIF-1 signaling in BM-EPCs. **a** Application of cortisol (COR) and dexamethasone (DEX) upon BM-EPCs and detection of mTOR activity indicators. **b** Application of COR at 1.0 μM and detection of mTOR-related genes. **c** Application of COR at 1.0 μM and detection of HIF-1-related genes (*n* = 3, *P < 0.05, **P < 0.01)

The lncRNA array showed that there were 188 genes with mRNA upregulation and 125 genes with mRNA downregulation (Fig. 1a). Also, there were 156 upregulated and 48 downregulated lncRNAs. The array yielded 17 significantly altered pathways annotated by KEGG (Fig. 2b–c). The mammalian target of rapamycin (mTOR) pathway was significantly downregulated in DEX-treated rats (*P* = 0.04996). Following the mTOR alteration was the downregulation of HIF-1 signaling with a marginal statistical significance. Given the knowledge-based angiogenesis regulatory pathway putting mTOR upstream of HIF-1 signaling, it was hypothesized that initial mTOR inhibition by glucocorticoids in ANFH exerted anti-angiogenic effect via HIF-1 signaling.

With isolated and cultured BM-EPCs, the phosphorylation of S6K was significantly suppressed following cortisol (COR) or DEX treatment and the suppression was dose-dependent (Fig. 2a). Using COR or DEX at 1.0 μM, significant downregulation of phospho-Akt (p-Akt) and phospho-eIF4B (p-eIF4b) were observed. No effect was seen on expression of FoxO3, p-FoxO3, p-PTEN, p-mTOR, or Raptor (Fig. 2b). The HIF-1-related genes were further pursued, and it was found that COR or DEX at 1.0 μM significantly downregulated HIF-1α and VEGFA expression, yet having no effect on HIF-1β, p300, or PHD (Fig. 2c).

**Fig. 3:**
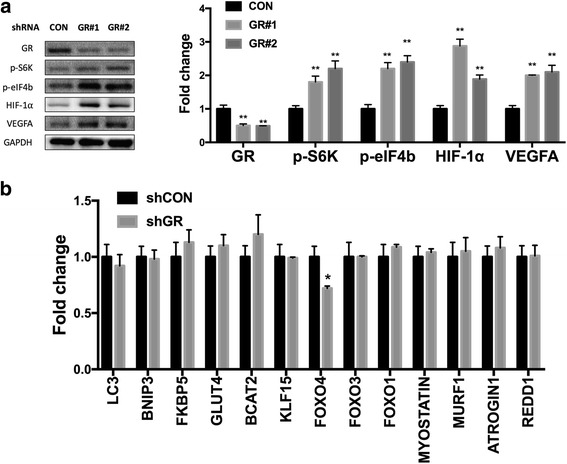
Impact of glucocorticoid receptor (GR) on mTOR/HIF genes in BM-EPCs. **a** Knockdown of GR and expression of mTOR- and HIF-related genes. **b** Knockdown of GR and expression of muscular atrophy-related genes (*n* = 3, *P < 0.05, **P < 0.01)

Whether counteracting the glucocorticoid receptor (GR) gene (NR3C1) could reverse the mTOR and subsequent HIF-1 signaling was the next to determine. By knocking down (KD) GR in BM-EPCs, significantly upregulated p-S6K, p-eIF4b, HIF-1α, and VEGFA were found (Fig. 3a). GR was also involved in muscle atrophy [9]. Whether atrophy-related genes were also altered in response to GR in ANFH was therefore studied. Detected by quantitative PCR, KD of GR did not alter expression of any other atrophy-related genes in BM-EPCs except for FoxO4 (Fig. 3b).

**Fig. 4:**
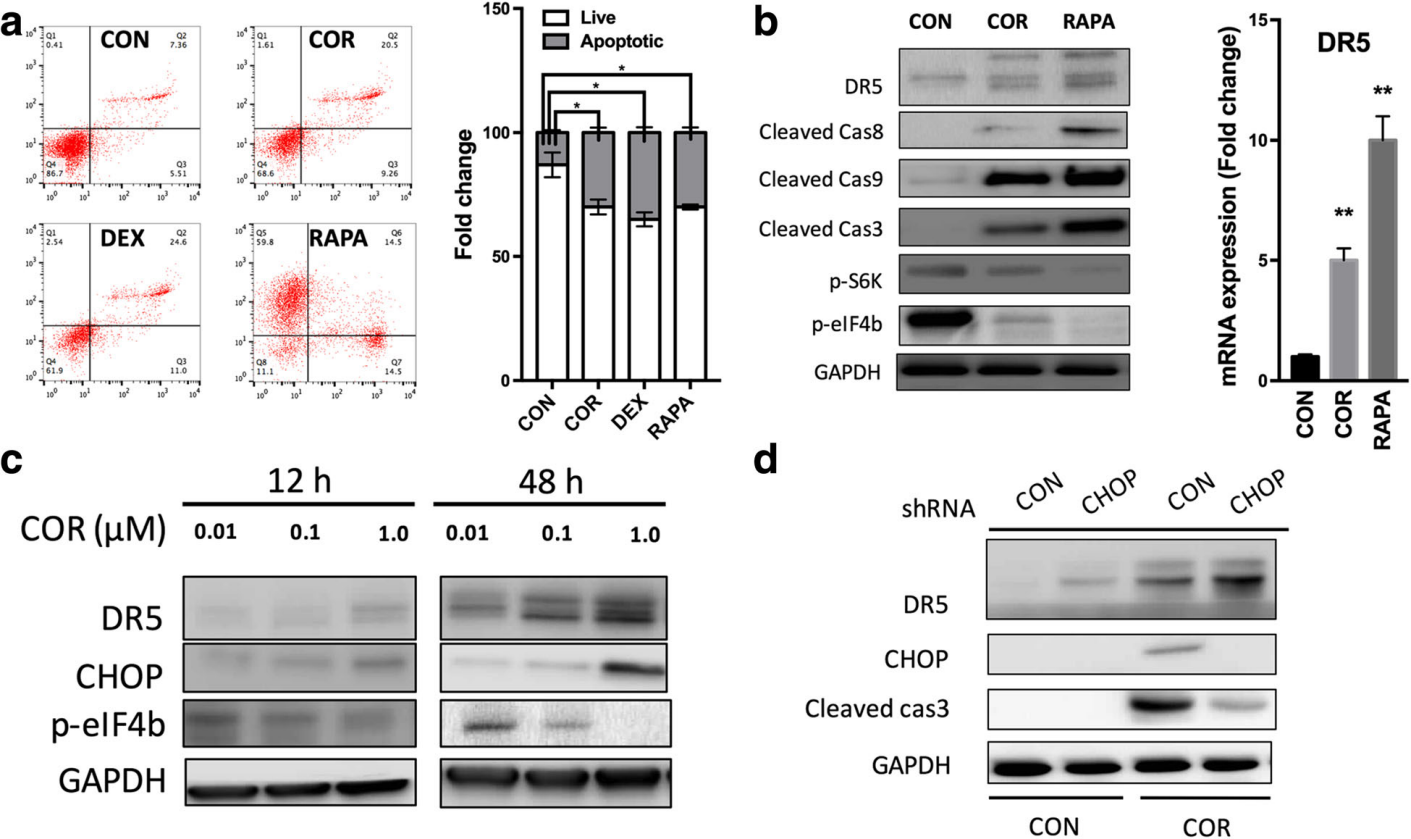
Inhibition of mTOR by cortisol-induced cell apoptosis in BM-EPCs. **a** Application of COR (1.0 μM), DEX (1.0 μM), and rapamycin (RAPA, 10 μM) inducing apoptosis. **b** Application of COR and RAPA impacting on expression of apoptosis and mTOR-related genes. **c** Application of COR impacting on expression of DR5 and CHOP. **d** KD of CHOP impacting on DR5 expression and apoptotic indicator (*n* = 3, *P < 0.05, **P < 0.01)

mTOR signaling was reported to play a role in cell apoptosis [10]. Whether inhibition of mTOR also induced apoptosis of BM-EPCs was then studied. COR or DEX at 1.0 μM, as well as rapamycin (RAPA) at 10 μM, induced significantly more apoptotic cells (Fig. 4a). Both COR and RAPA induced significant upregulation of DR5 and cleaved caspase 3, 9, and 8, together with suppression of mTOR activity indicators (Fig. 4b). Alteration of DR5 in BM-EPCs in response to COR was in accordance with alteration of CHOP and both alterations were dose dependent (Fig. 4c). To determine the regulatory role between CHOP and DR5, CHOP KD was applied and found that without CHOP, the pro-apoptotic effect of COR was mitigated, indicated by cleaved caspase 3 level (Fig. 4d).

## Discussion

In the current study, glucocorticoid-induced ANFH via inhibition of mTOR signaling was found, a mechanism not reported previously. Detailed regulatory mechanism included impaired angiogenesis by subsequent HIF-1 inhibition and increased CHOP-dependent DR5-mediated apoptosis. Those findings offered novel perspective on the impact of glucocorticoids on ANFH.

Role of impairment of angiogenesis in ANFH has been emphasized. It is reported that circulating endothelial progenitor cell (EPC) numbers and functions are reduced in ANFH patients, suggesting that risk factors of ANFH may alter EPCs biology in angiogenesis and vascular repair [11]. Further, recombinant adeno-associated viral vectors co-expressing the hVEGF(165) and hBMP(7) genes showed efficient gene expression ability on angiogenesis. The VEGF(165) and BMP(7) proteins expressed from the vector have efficient biological activity in vitro [12]. Small intestinal submucosae matrix (SIS) is composed of highly conserved collagens, glycoproteins, proteoglycans, and glycoaminoglycans in their natural configuration and concentrations. Transplantation of PBSCs cultured with SIS effectively improved ischemic femoral head necrosis with significant increase of new blood vessels [13]. Co-culture of mesenchymal stem cells with umbilical vein endothelial cells also showed increased cell survival, migration, and angiogenesis, with upregulation of expression of SDF-1α, VEGF, and IL-6 expression [14]. Those pilot studies pushed forward stem cell investigation in human studies. Intravenous transplantation of allogeneic MSCs can promote vascular and bone regeneration in the necrotic region of the femoral head in a rabbit model of ANFH. The results of our study suggest that the intravenous transplantation of MSCs could be a potential and minimally invasive treatment option for ANFH patients [15]. Interestingly, a novel calcium phosphate (CPC) composite scaffold, which contains BMP-VEGF-loaded poly-lactic-co-glycolic acid (PLGA) microspheres (BMP-VEGF-PLGA-CPC) was generated and implanted into the bone tunnels of core decompression in the femoral head, showing the scaffold may improve the therapeutic effect of core decompression surgery and be used as a treatment for ANFH [16]. Those studies provide compelling evidence that angiogenesis plays a critical role in ANFH recovery, and our results indicate that enhancement of mTOR could be another treatment modality. One of the critical findings of the current study was inhibition of mTOR signaling also induced apoptosis of BM-EPCs via CHOP-dependent DR5 induction in response to GR stimulation. This finding is in line with the study by Shimizu et al. [9], in which authors found that mTOR activation inhibits GR transcription function and efficiently counteracts the catabolic processes provoked by glucocorticoids in skeletal muscle tissue. Other scholars find that rapamycin sensitizes glucocorticoid-resistant acute lymphoblastic leukemia CEM-C1 cells to dexamethasone-induced apoptosis through both mTOR suppression and upregulation and activation of glucocorticoid receptor [17]. The main risk factors are bone fractures, joint dislocations, alcoholism, and the use of high-dose steroids. Other risk factors include radiation therapy, chemotherapy, and organ transplantation. Unlike mechanic factors that directly disrupt the blood supply of the femoral head, etiology for corticosteroid-induced ANFH remains unclear. Most suggest that the drugs may interfere degradation of lipids, which accumulate and clog the vessels, eventually leading to avascularity. This phenotype is more severe as it is more frequently to affect both hips. Given that mTOR signaling is strongly associated with lipid metabolism, the mTOR-GR axis appears to be a promising target for the disease management.

Combating apoptosis of progenitor cells is another target in treating ANFH. It was reported that The Wnt/β-catenin pathway is involved in the pathogenesis of early stage ANFH in rat model, and it may act through the regulation of c-Myc, which affects the cell cycle and cell apoptosis [18]. Alternatively, cell death was used to trace early evidence of ANFH. It was shown that (99m)Tc-annexin V is superior to (99m)Tc-MDP for the early detection of glucocorticoid-induced femoral head necrosis in the rabbit and may be a better strategy for the early detection of glucocorticoid-induced femoral head necrosis in patients [19]. One group reported that Chinese herb, Tao-Hong-Si-Wu decoction (THSWD), significantly promoted the expression of HIF-1α and VEGF in the femoral head tissue of rabbits and markedly inhibit the apoptosis of osteocytes, chondrocytes, and bone marrow cells. In addition, THSWD suppressed caspase-3 expression and induced bcl-2 expression in femoral head tissues. Like in our study, they concluded that THSWD can suppress ANFH by regulating the HIF-1α pathway and cell apoptosis [20]. Another study pointed out that was able to reduce steroid-induced bone cellular apoptosis, reduce the occurrence of necrosis of the femoral head and, through in vivo metabolism, it may promote the synthesis and release of IGF-1 [21]. More commonly accessible drug, vitamin E was also reported to benefit ANFH patients via combating apoptosis. Report showed that vitamin E is effective in intervening in apoptosis through decreasing caspase-3 expression and upregulating Bcl-2 expression and by alleviating DNA oxidative damage in bone marrow hemopoietic cells at the early stage of steroid-induced femoral head necrosis in rabbit models [22]. So far, no study focused on apoptosis of BM-EPC, the direct progenitor for angiogenesis. Our results for the first time revealed that the apoptosis induced by excessive steroid also included endothelial progenitors, and combined impairment of osteocytes and angiogenesis contributes to the pathogenesis.

## Conclusion

Insightful understanding of the molecular alteration in ANFH could contribute to the therapeutic strategy. We, here, showed that decreased mTOR signaling in response to GR stimulation led to downregulated HIF activity and increased apoptosis, which could be, in part, the pathophysiology. Our findings indicate that enhancing mTOR signaling could be actionable and of therapeutic potential in ANFH.

## Additional files

Additional file 1: Raw normalized data of LncRNA microarray. (XLSX 25998 kb)

Additional file 2: Table S1. Antibodies list used for Western blot. **Table S2.** Oligonucleotides list used for real-time PCR. (DOCX 14 kb)

## Abbreviations

ANFH: Avascular necrosis of the femoral head
BM-EPCs: Bone marrow-endothelial progenitor cells
EPCs: Circulating endothelial progenitor cells
mTOR: Mammalian target of rapamycin

## References

[1] Microsurgery Department of the Orthopedics Branch of the Chinese Medical Doctor A, Group from the O, Bone Defect Branch of the Chinese Association of R, Reconstructive S, Microsurgery, Reconstructive Surgery Group of the Orthopedics Branch of the Chinese Medical A (2017). Chinese guideline for the diagnosis and treatment of osteonecrosis of the femoral head in adults. Orthop Surg.

[2] Guerado E, Caso E (2016). The physiopathology of avascular necrosis of the femoral head: an update. Injury.

[3] Tsertsvadze A, Grove A, Freeman K, Court R, Johnson S, Connock M, Clarke A, Sutcliffe P (2014). Total hip replacement for the treatment of end stage arthritis of the hip: a systematic review and meta-analysis. PLoS One.

[4] Bilge O, Doral MN, Yel M, Karalezli N, Miniaci A (2015). Treatment of osteonecrosis of the femoral head with focal anatomic-resurfacing implantation (HemiCAP): preliminary results of an alternative option. J Orthop Surg Res.

[5] Wang F, Wang Y, Hu N, Miao X (2016). Risk-factors, pathogenesis, and pharmaceutical approaches for treatment of steroid-induced bone infarction of femoral head. Acta Pol Pharm.

[6] Roth A, Beckmann J, Bohndorf K, Fischer A, Heiss C, Kenn W, Jager M, Maus U, Noth U, Peters KM (2016). S3-guideline non-traumatic adult femoral head necrosis. Arch Orthop Trauma Surg.

[7] Chen JK, Deng YP, Jiang GJ, Liu YZ, Zhao T, Shen FM (2013). Establishment of tube formation assay of bone marrow-derived endothelial progenitor cells. CNS Neurosci Ther.

[8] Feng C, Sun Y, Ding G, Wu Z, Jiang H, Wang L, Ding Q, Wen H (2015). PI3Kbeta inhibitor TGX221 selectively inhibits renal cell carcinoma cells with both VHL and SETD2 mutations and links multiple pathways. Sci Rep.

[9] Shimizu N, Yoshikawa N, Ito N, Maruyama T, Suzuki Y, Takeda S, Nakae J, Tagata Y, Nishitani S, Takehana K (2011). Crosstalk between glucocorticoid receptor and nutritional sensor mTOR in skeletal muscle. Cell Metab.

[10] He K, Zheng X, Li M, Zhang L, Yu J (2016). mTOR inhibitors induce apoptosis in colon cancer cells via CHOP-dependent DR5 induction on 4E-BP1 dephosphorylation. Oncogene.

[11] Feng Y, Yang SH, Xiao BJ, WH X, Ye SN, Xia T, Zheng D, Liu XZ, Liao YF (2010). Decreased in the number and function of circulation endothelial progenitor cells in patients with avascular necrosis of the femoral head. Bone.

[12] Shi ZB, Wang KZ (2010). Effects of recombinant adeno-associated viral vectors on angiopoiesis and osteogenesis in cultured rabbit bone marrow stem cells via co-expressing hVEGF and hBMP genes: a preliminary study in vitro. Tissue Cell.

[13] Song HJ, Lan BS, Cheng B, Zhang KF, Yan HW, Wang WZ, Gao ZQ (2011). Treatment of early avascular necrosis of femoral head by small intestinal submucosal matrix with peripheral blood stem cells. Transplant Proc.

[14] Zhang B, Yang S, Zhang Y, Sun Z, Xu W, Ye S (2012). Co-culture of mesenchymal stem cells with umbilical vein endothelial cells under hypoxic condition. J Huazhong Univ Sci Technolog Med Sci.

[15] Li Z, Liao W, Zhao Q, Liu M, Xia W, Yang Y, Shao N (2013). Angiogenesis and bone regeneration by allogeneic mesenchymal stem cell intravenous transplantation in rabbit model of avascular necrotic femoral head. J Surg Res.

[16] Zhang HX, Zhang XP, Xiao GY, Hou Y, Cheng L, Si M, Wang SS, Li YH, Nie L (2016). In vitro and in vivo evaluation of calcium phosphate composite scaffolds containing BMP-VEGF loaded PLGA microspheres for the treatment of avascular necrosis of the femoral head. Mater Sci Eng C Mater Biol Appl.

[17] Guo X, Zhou CY, Li Q, Gao J, Zhu YP, Gu L, Ma ZG (2013). Rapamycin sensitizes glucocorticoid resistant acute lymphoblastic leukemia CEM-C1 cells to dexamethasone induced apoptosis through both mTOR suppression and up-regulation and activation of glucocorticoid receptor. Biomed Environ Sci.

[18] Zhang C, Zou YL, Ma J, Dang XQ, Wang KZ (2015). Apoptosis associated with Wnt/beta-catenin pathway leads to steroid-induced avascular necrosis of femoral head. BMC Musculoskelet Disord.

[19] Wang X, Liu Y, Wang X, Liu R, Li J, Zhang G, Li Q, Wang L, Bai Z, Zhao J (2016). The role of (99m)Tc-annexin V apoptosis scintigraphy in visualizing early stage glucocorticoid-induced femoral head osteonecrosis in the rabbit. Biomed Res Int.

[20] Wu J, Yao L, Wang B, Liu Z, Ma K (2016). Tao-Hong-Si-Wu decoction ameliorates steroid-induced avascular necrosis of the femoral head by regulating the HIF-1alpha pathway and cell apoptosis. Biosci Trends.

[21] Xi H, Tao W, Jian Z, Sun X, Gong X, Huang L, Dong T (2017). Levodopa attenuates cellular apoptosis in steroid-associated necrosis of the femoral head. Exp Ther Med.

[22] Jia YB, Jiang DM, Ren YZ, Liang ZH, Zhao ZQ, Wang YX (2017). Inhibitory effects of vitamin E on osteocyte apoptosis and DNA oxidative damage in bone marrow hemopoietic cells at early stage of steroid-induced femoral head necrosis. Mol Med Rep.

