# Supplementary material for: Cortisol inhibits mTOR signaling in avascular necrosis of the femoral head
Source: J Orthop Surg Res. 2017 Oct 18;12:154. doi: 10.1186/s13018-017-0656-2 (PMC5648506; doi:10.1186/s13018-017-0656-2)
Supplement: Supplementary file 2 — Antibodies list used for Western blot. Table S2. Oligonucleotides list used for real-time PCR. (DOCX 14 kb) [file 13018_2017_656_MOESM2_ESM.docx]

**Table S1.** Antibodies list used for Western blot

| Protein name | Company | Catalog number |
| --- | --- | --- |
| HIF-1α | R&D System | MAB1536 |
| HIF-1β | Abcam | ab2771 |
| p300 | ThermoFisher | MA1-16608 |
| VEGFA | Abcam | ab51745 |
| PHD | Novus Biologicals | NB100-310 |
| GR | Proteintech | 24050-1-AP |
| DR5 | Abcam | ab8416 |
| Caspase 8 | R&D System | AF1650 |
| Caspase 3 | Abcam | ab52293 |
| Caspase 9 | Abcam | ab25758 |
| CHOP | Proteintech | 15204-1-AP |
| AKT | Cell signaling technology | 4691 |
| p-AKT | Cell signaling technology | 4060 |
| S6K | Cell signaling technology | 2708 |
| p-S6K | Cell signaling technology | 9234 |
| p-S6 | Cell signaling technology | 4858 |
| p-eIF4B | Cell signaling technology | 3591 |
| FoxO3 | Santa cruz biotechnology | 11351 |
| p-FoxO3 | Santa cruz biotechnology | 101683 |
| mTOR | Cell signaling technology | 2983 |
| p-mTOR | Cell signaling technology | 5536 |
| Raptor | Cell signaling technology | 2280 |
| GAPDH | Abcam | ab9485 |
| Anti-rabbit igG, HRP linked | Cell signaling technology | 7074 |

**Table S2. Oligonucleotides list used for real-time PCR**

| Name | Sequence (5’ to 3’) |
| --- | --- |
| GAPDH | AGGTCGGTGTGAACGGATTTG  GGGGTCGTTGATGGCAACA |
| LC3 | GACCGCTGTAAGGAGGTGC  CTTGACCAACTCGCTCATGTTA |
| BNIP3 | CTGGGTAGAACTGCACTTCAG  GGAGCTACTTCGTCCAGATTCAT |
| FKBP5 | GATGAGGGCACCAGTAACAATG  CAACATCCCTTTGTAGTGGACAT |
| GLUT4 | ACACTGGTCCTAGCTGTATTCT  CCAGCCACGTTGCATTGTA |
| BCAT2 | ACAGACCACATGCTGATGGTG  CTGGGTGTAGCGTGAGGTTC |
| KLF15 | GGCAGTGGAGGTATTGGAGAT  GGTCCCTGCTACCGTTCTCT |
| FOXO1 | GGCGGGCTGGAAGAATTCAA  GCCTCCCTCTGGATTGAGCA |
| FOXO3 | GAGTCCCCTCGTCGCGGT  GAGCTCCAGCTCGGCTCCTTC |
| FOXO4 | CTTCCTCGACCAGACCTCG  ACAGGATCGGTTCGGAGTGT |
| MYOSTATIN | AGTGGATCTAAATGAGGGCAGT  GGAGTACCTCGTGTTTTGTCTC |
| MURF1 | CCAGGCTGCGAATCCCTAC  ATTTTCTCGTCTTCGTGTTCCTT |
| ATROGIN1 | CAGCTTCGTGAGCGACCTC  GGCAGTCGAGAAGTCCAGTC |
| REDD1 | GTGCTGCGTCTGGACTCTC  CCGGTACTTAGCGTCAGGG |
